# QTLs for earliness and yield-forming traits in the Lubuski × CamB barley RIL population under various water regimes

**DOI:** 10.1007/s13353-016-0363-4

**Published:** 2016-08-09

**Authors:** Piotr Ogrodowicz, Tadeusz Adamski, Krzysztof Mikołajczak, Anetta Kuczyńska, Maria Surma, Paweł Krajewski, Aneta Sawikowska, Andrzej G. Górny, Kornelia Gudyś, Iwona Szarejko, Justyna Guzy-Wróbelska, Karolina Krystkowiak

**Affiliations:** 1 Institute of Plant Genetics, Polish Academy of Sciences, Strzeszyńska 34, 60-479 Poznań, Poland; 2 Department of Genetics, Faculty of Biology and Environmental Protection, University of Silesia, Jagiellońska 28, 40-032 Katowice, Poland

**Keywords:** Consensus map, Drought, Earliness, SNP annotation, SNP markers, Spring barley, SSR markers

## Abstract

Drought has become more frequent in Central Europe causing large losses in cereal yields, especially of spring crops. The development of new varieties with increased tolerance to drought is a key tool for improvement of agricultural productivity. Material for the study consisted of 100 barley recombinant inbred lines (RILs) (LCam) derived from the cross between Syrian and European parents. The RILs and parental genotypes were examined in greenhouse experiments under well-watered and water-deficit conditions. During vegetation the date of heading, yield and yield-related traits were measured. RIL population was genotyped with microsatellite and single nucleotide polymorphism markers. This population, together with two other populations, was the basis for the consensus map construction, which was used for identification of quantitative trait loci (QTLs) affecting the traits. The studied lines showed a large variability in heading date. It was noted that drought-treatment negatively affected the yield and its components, especially when applied at the flag leaf stage. In total, 60 QTLs were detected on all the barley chromosomes. The largest number of QTLs was found on chromosome 2H. The main QTL associated with heading, located on chromosome 2H (Q.HD.LC-2H), was identified at SNP marker 5880–2547, in the vicinity of *Ppd-H1* gene. SNP 5880–2547 was also the closest marker to QTLs associated with plant architecture, spike morphology and grain yield. The present study showed that the earliness allele from the Syrian parent, as introduced into the genome of an European variety could result in an improvement of barley yield performance under drought conditions.

**Electronic supplementary material:**

The online version of this article (doi:10.1007/s13353-016-0363-4) contains supplementary material, which is available to authorized users.

## Introduction

Barley (*Hordeum vulgare* L.) is not only one of the most important crops from an economic point of view (FAOSTAT 2014), but it is also an excellent species for genome mapping and map-based analyses (Costa et al. 2001; Mansour et al. 2014). Its diploid nature, low chromosome number and a high degree of self-fertility mean that barley is a common subject for genetic studies examining drought resistance of crops (Tondelli et al. 2006; Talamè et al. 2007).

Several genetic maps based upon different genetic marker techniques have been published (Wenzl et al. 2006; Zhou et al. 2015). Among various types of DNA markers, microsatellites (SSR) and single nucleotide polymorphism (SNP) have been widely used for genome analyses (Cockram et al. 2010; Cuesta-Marcos et al. 2010; Honsdorf et al. 2014; Varshney et al. 2007). The first high-density gene map based on SNP markers contained 2.943 SNP loci in 975 marker bins and covered a genetic distance of 1099 cM (Close et al. 2009). The availability of high-throughput SNP genotyping has facilitated the genetic studies of agronomically important traits (Close et al. 2004; Wang et al. 2010b). Recently, research containing a detailed overview of the functional portions of the barley genome has been published (International Barley Genome Sequencing Consortium 2012). This highly resoluted genetic map together with the sequence data has a tremendous potential for candidate gene discovery using conservation of the grass genome synteny (Mayer et al. 2009).

Abiotic stresses reduce average yields for most crops (Boyer 1982; Bray 1997). Among the stresses, water deficit is the most devastating on a global scale (Zhao and Runnings 2009). Aspinall et al. (1964) and Samarah (2005) have reported stage-specific drought responses in the crops. Appropriate irrigation conditions during the stem elongation phase are indispensable for the formation of fertile florets at anthesis as the final number of grains is determined during this period (Miralles and Slafer 1995). Water deprivation in this critical developmental stage affects numerous aspects of plant metabolism leading to impairment of many biochemical pathways (Moran et al. 1994; Loggini et al. 1999; Farooq et al. 2009).

The simplest solution to survive in dry environments is an escape from drought (Passioura 1996; Richards 1996). The short life cycle of crop plants might be considered as an important trait related to water deficit adaptation (Araus et al. 2002). The majority of barley cultivars vary significantly in their response to water scarcity (Zare 2012). Sources of drought tolerance can be found in landraces from geographical regions with challenging climates close to their domestication origin (Ellis et al. 2000; Górny 2001; Nevo and Chen 2010).

Heading date in barley depends on vernalisation requirements (Takahashi and Yasuda 1956; Sasani et al. 2012), photoperiodic response (Roberts et al. 1988; Laurie et al. 1995) and earliness *per se* genes (Gallagher et al. 1991; Sameri et al. 2006). Quantitative trait loci (QTLs) associated with heading date have been mapped on all barley chromosomes (e.g. Hayes et al. 1993; Laurie et al. 1995; Tinker et al. 1996; Bezant et al. 1997; Qi et al. 1998; Pillen et al. 2003) and many allele-specific markers for some candidate genes controlling these processes are known (Turner et al. 2005; Faure et al. 2007; Szűcs et al. 2007).

The major photoperiod response locus has been identified by RFLP analysis on the short arm of chromosome 2H. Dominant alleles at *Ppd-H1* accelerate flowering under long day conditions, whereas no effect has been detected under short day conditions. Laurie et al. (1995) and Turner et al. (2005) have shown that the late-flowering allele is recessive. Two main single nucleotide polymorphisms have been detected which differentiate alleles involved in the plant sensitivity to day length. Non-synonymous (G – *Ppd-H1*/ A – *ppd-h1*) SNP within the CCT domain has been suggested as an explanation for recessive form of the allelic variation (Turner et al. 2005). Another study revealed that a polymorphism in the photoperiodic response in barley varieties might be associated with the SNP48 situated in the exon 6 of the *Ppd-H1* coding region) (Jones et al. 2008). A second major photoperiodic response locus (*Ppd-H2*) has been mapped to the chromosome 1H (Laurie et al. 1995). The *Ppd-H2* affects the flowering time under short day conditions. A candidate gene (*HvFT3*) for this locus has been proposed by Faure et al. (2007).

The vernalisation and photoperiodic pathways correspond to each other to promote flowering in crops (Distelfeld et al. 2009). A study conducted using barley spring crosses revealed loci for flowering time in the regions connected with vernalisation response (Bezant et al. 1997). Three genes, *Vrn-H1*, *Vrn-H2* and *Vrn-H3*, located on the chromosomes 5H, 4H and 7H, respectively, have been proposed as the major vernalisation response genes (Cockram et al. 2007).

The aim of the present study was to detect QTLs determining yield and yield-forming traits in a recombinant inbred line (RIL) population developed from a hybrid between European and Syrian genotypes (adapted to dry environments) under optimal and water stress conditions, with special attention being paid to earliness.

## Materials and methods

### Plant material

Material for the study covered RIL population of spring barley (*Hordeum vulagre* L.) derived from the cross Lubuski × Cam/B1/CI08887//CI05761. The parent Cam/B1/CI08887//CI05761 (hereafter referred as CamB) is the Syrian breeding line supplied to Dr. A. Górny by Drs S. Grando and S. Ceccarelli from ICARDA in Aleppo and Lubuski is an old Polish cultivar derived from a Heines-Haisa/Skrzeszowicki hybrid. The examined population was developed by means of the single seed descent (SSD) technique (up to F_8_) (Goulden 1939) associated with *in vitro* culture of immature embryos (Surma et al. 2013). Out of 150 developed RILs 100 were randomly chosen for the present experiments.

### Greenhouse experiments

The greenhouse experiments with the Lubuski × CamB population were conducted (was grown in three replicates) in two growing seasons (2012, 2013) during April–August. In both years, three water regimes were applied: (1) C – optimal water supply for the whole vegetation period, (2) DI – drought stress beginning at the three-leaf stage (13 in the BBCH scale) and maintained for 10 days, (3) DII – drought stress beginning at the flag leaf stage (37 in the BBCH scale) and maintained for 14 days, which created six environments denoted as: C 2012, C 2013, DI 2012, DI 2013, DII 2012 and DII 2013. Ten plants were grown in pots containing 9 kg of soil. Air moisture and temperature were monitored by special device (LOG32 - Temperature-humidity logger with integrated USB-interface and automatic PDF-creation). Control of the soil moisture was provided by a hand-held device (FOM/mts) designed for field measurements of the soil moisture and temperature (Malicki et al. 1996). The weighing method was used as an additional control of the irrigation system. The soil moisture was kept at 2.2 and 3.2 pF in optimal and drought conditions, respectively (ESM 1). Three groups of traits were observed: associated with morphology of the main and lateral spikes (grain weight per main spike - GWSm, number of grains per main spike - NGSm, number of spikelets per main spike - NSSm, length of main spike - LSm, grain weight per lateral spike - GWSl, number of grains per lateral spike - NGSl, number of spikelets per lateral spike- NSSl, length of lateral spike - LSl), with plant architecture (length of main stem - LSt, number of productive tillers per plant - NPT), and with grain yield (1000-grain weight - TGW, Grain weight per plant - GWP). Duration of the vegetative growth period was expressed as the number of days from sowing to heading (heading date - HD). The measured traits are listed in ESM 2.

### Genotyping

In the present studies consensus map constructed by Mikołajczak et al. (2016) was used for QTL analysis. Briefly: A set of 78 barley SSR markers developed by Varshney et al. (2007) was used in the experiment. SNP genotyping was carried out at the Southern California Consortium using the Illumina GoldenGate array 1 (Illumina Inc., San Diego, CA) that analyses 1.536 genome-wide single nucleotide polymorphisms; details of this array (BOPA - barley oligo pool assays) are described by Close et al. (2009).

JoinMap 3.0 software (Van Ooijen and Voorrips 2001) was used for the map construction. Once the individual genetic map was obtained, the consensus map was constructed. The complete dataset consisted of 819 markers mapped in the Maresi × CamB (MCam), Lubuski × CamB (LCam) and Georgie × Harmal (GH) populations. Details on the development of the map construction are given in Mikołajczak et al. (2016).

### Statistical analysis

Observations for RILs were processed by analysis of variance in a mixed model with fixed effects for year, drought and year × drought interaction, and with random effects for line and interaction of line with year and/or drought treatment. The residual maximum likelihood (REML) algorithm was used to estimate variance components for random effects and the *F*-statistic was computed to assess the significance of the fixed effects. Ordinary, mean values computed for RILs in all specific (years × drought) - combinations were used for construction of principal component biplots. Pearson correlation coefficients between all the analysed traits were calculated. QTL analysis was performed for the consensus linkage map (Mikołajczak et al. 2016) with the mixed-model approach described by Malosetti et al. (2013), including optimal genetic correlation structure selection and the significance threshold estimation. The interval mapping was conducted with a step size of 2 cM by selecting the QTL candidate and then using them iteratively as cofactors until the list of QTL was not changed. The threshold for the − log10(P-value) statistic was computed by the method of Li and Ji (2005) to ensure the genome-wide error rate was less than 0.01. The windows for not selecting two close QTLs and for exclusion of cofactors were set at 10 and 30 cM, respectively. Selection of the set of QTL effects in the final model was performed at *P <* 0.05; the *P*-values for the Wald test were computed as the mean from the values obtained by adding and dropping the QTL main and interaction effects in the model. All the above computations were performed in Genstat 16 (VSN Int. 2013).

### QTL annotation

All SNP sequences taken from Close et al. (2009) (Supplementary material file BOPA1 SNP 1471-2164-10-582-S19.xls) were mapped using NCBI Blast for Windows to barley genomic space in Ensembl Plants ver. 2.28 (reference repeat masked sequence Hordeum_vulgare.082214v1.28.dna_rm.toplevel.fa, maximum EValue = 1e-060, minimum 95 % identity of the SNP sequence). The SNP mapping positions were used to obtain a projection of two LOD QTL support intervals (see Xu 2010) onto the genomic sequence; all genes located in projected intervals were listed and annotated using Gene Ontology (GO) terms. For QTL interpretation, we applied a method similar to the one implemented by Cantalapiedra et al. (2015).

### Early and late heading subgroups of plants

According to SNP 5880–2547 segregation (Mansour et al. 2014; Muñoz-Amatriaín et al. 2011), RILs were divided into two subgroups—early heading (group A – allele from CamB) and late heading plants (group B – allele from Lubuski).

## Results

### Phenotypic evaluation

The average heading dates and the mean values of morphological traits for parental cultivars and RILs in the six environments are presented in ESM 3a and ESM 3b as supplementary material, respectively. Parental genotypes are classified as early (CamB) and late (Lubuski) according to the large differences between their heading dates in all experiments. The heading (HD) of the Syrian genotype grown under well-watered conditions was about 15–19 days earlier than of the European one. The HD for parents increased both in DI conditions (by about 3 - 6 days) and in DII conditions (by about 1 - 6 days) as compared to the well-watered conditions. The data analysis across two years showed highly significant differences among lines for HD. RILs with longer vegetation periods than the late-heading parent were noticed among the studied population in all environments. For all studied traits, Lubuski showed significantly higher values under well-watered conditions compared to CamB (with the exception of LSt in 2012, 2013 and NPT in 2013). The Syrian cultivar showed higher values for TGW than the European parent in DI over the two years. On the other hand, Lubuski showed higher GWP in all environments. The comparison of the parental genotypes for traits connected with the plant architecture showed that CamB formed more productive tillers under water-stress conditions applied at the three-leaf stage.

In RILs, NPT increased both under drought I and drought II conditions. For all observed traits (with the exceptions: HD and NPT), greater decrease were noticed under drought II.

Lines of the LCam population were significantly differentiated in terms of all analysed traits (Table 1). In all case, the variance components for all types of interactions were smaller than that for lines. For HD, variance components were significant for all types of interaction (i.e. for line × year, line × water regimes, line × year × water regimes). On the other hand, no interaction component was significant for NGSm and LSl.

**Table 1:** ANOVA results and variance components estimates for agronomic traits observed in LCam population

Trait (abbrev.)	P-values for significance of effects of			Variance components and std. errors for							
	years (Y)	treatment (D)	Y × D interaction	lines	s.e.	interaction line x year	s.e.	interaction line x treatment	s.e.	interaction line x year x treatment	s.e.
HD	<0.001	< 0.001	< 0.001	11.8878*	2.4091	1.8182*	0.5891	8.3332*	1.1899	6.0901*	0.6216
TGW	< 0.001	< 0.001	< 0.001	3.0051*	0.7274	1.4115*	0.4623	0.7955	0.4152	0.0146	0.5255
GWP	< 0.001	< 0.001	< 0.001	0.0327*	0.0076	0.0216*	0.005	0.0013	0.0028	0.0005	0.004
LSt	< 0.001	< 0.001	< 0.001	18.5399*	3.3819	5.0418*	1.3247	0.7975	0.8817	1.3372	1.2274
NPT	< 0.001	< 0.001	< 0.001	0.0817*	0.0193	0.0075	0.01	0.0476*	0.0154	0.0228	0.0167
GWSm	< 0.001	< 0.001	< 0.001	0.0073*	0.0013	0.0007	0.0003	0.0018*	0.0004	0.0008	0.0004
NGSm	< 0.001	< 0.001	< 0.001	4.2621*	0.665	0.1455	0.0939	0.3227	0.1172	0.2625	0.1393
NSSm	< 0.001	< 0.001	< 0.001	5.1929*	0.7881	0.1276	0.0845	0.2369	0.1022	0.387*	0.1242
LSm	< 0.001	< 0.001	< 0.001	0.4565*	0.0709	0.0121	0.0097	0.0326	0.0126	0.06*	0.0147
GWSl	< 0.001	< 0.001	< 0.001	0.0031*	0.0007	0.0013*	0.0004	0	0.0003	0.0008	0.0004
NGSl	< 0.001	< 0.001	< 0.001	1.6608*	0.365	1.0565*	0.2214	0.0386	0.1002	0.2787	0.1471
NSSl	< 0.001	< 0.001	< 0.001	3.3928*	0.5479	0.4478*	0.1179	0.1225	0.0816	0.0809	0.1104
LSl	< 0.001	< 0.001	< 0.001	0.31*	0.0494	0.0223	0.0092	0.0191	0.0093	0.0173	0.0116

* variance component at least three times greater than its standard error

s.e.- standard error

**Fig. 1:**
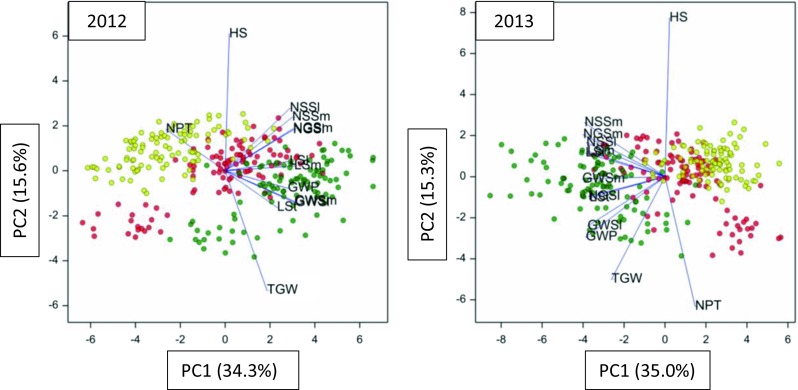
Principal component biplots, with dots corresponding to LCam recombinant inbred lines observed in drought DI (*red*), drought DII (*yellow*) and in control conditions (*green*), and vectors corresponding to observed traits, made for data obtained in 2012 and 2013

As shown in biplots (Fig. 1), RIL plants grown in drought II were affected more than in drought I, as they are further away from control plants superior—in both years—by spike morphology traits and LSt.

Correlations of all traits with HD were significant (*P* < 0.001) in at least one environment, with no correlation significant in DII 2012 (Table 2). The highest correlation coefficient was found for NSSl in drought I in 2012 (r = 0.762), whereas the correlation between HD and NGSl (DII 2013) was the weakest (r = 0.236). Significant negative correlations between HD and NPT, revealed also in 2013 biplot, were observed across three environments (DI 2012, DI 2013, C 2013) which indicates that early heading lines developed more productive tillers, especially in DI conditions. No significant association was found between days to heading and 1000-grain weight, except for the control conditions in 2012 (negative correlation). Positive and significant correlations were recorded between HD and spike traits: GWSm, NGSm, NSSm, LSm, GWSl, NGSl, NSSl in both years in DI and C conditions. This indicates that late heading lines developed longer spikes with more spikelets and—as a consequence—more grains. Moreover, a positive correlation was found between GWP and heading stage, which indicates that early heading lines were characterised by lower yield.

**Table 2:** Correlation coefficients between HD and yield forming traits under well-watered and drought conditions

Trait	Treatment					
	DI 2012	DI 2013	DII 2012	DII 2013	C 2012	C 2013
TGW	n.s.	n.s.	n.s.	n.s.	−0.350	n.s.
GWP	0.520	0.295	n.s.	n.s.	0.438	0.296
LSt	n.s.	0.296	n.s.	n.s.	n.s.	n.s.
NPT	−0.583	−0.444	n.s.	n.s.	n.s.	−0.313
GWSm	0.658	0.523	n.s.	n.s.	0.536	0.413
NGSm	0.755	0.618	n.s.	n.s.	0.661	0.614
N.S.Sm	0.745	0.593	n.s.	n.s.	0.656	0.581
LSm	0.630	0.426	n.s.	n.s.	0.467	0.251
GWSl	0.627	0.357	n.s.	n.s.	0.326	0.299
NGSl	0.717	0.461	n.s.	0.236	0.548	0.456
NSSl	0.762	0.572	n.s.	0.321	0.534	0.575
LSl	0.597	0.490	n.s.	0.287	n.s.	0.402

n.s.- not significant

Correlations shown are significant at the P < 0.001 level

### QTL analyses

A total of 60 QTLs were detected on all chromosomes (Table 3). The largest number of QTLs were found on chromosome 2H (23 QTLs). Only three QTLs were detected on chromosome 1H. The largest number of QTLs were found for NGSm and LSm (nine QTLs). The lowest number of QTLs were found for GWP (one QTL). The QTL × E interaction was found for 68 % of QTLs detected. All QTLs for HD and NSSm showed QTL × E interaction (ESM 4).

**Table 3:** QTLs identified in the LCam population for the observed traits

Trait	QTL ID	Linkage group	Position (cM)	Marker	Synonim BOPA1	−Log10 (P-value) ^d)^	Shift from marker to QTL position (cM)	QTL x E (a)	Additive effect (b)						Percent of variance explained by QTL in years (%) (c)					
									DI	DII	C	DI	DII	C	DI	DII	C	DI	DII	C
									2012	2012	2012	2013	2013	2013	2012	2012	2012	2013	2013	2013
Heading date	Q.HD.LC-2H	2H	10.74	5880-2547	11_21015	57.33	0.00	1	−6.32	0.81	−6.41	−6.18	−0.99	−5.40	99.02	9.55	75.84	102.96	25.77	112.57
	Q.HD.LC-3H.1	3H.1	47.56	10353-119	11_10011	3.54	0.00	1	1.03	n.s.	2.01	n.s.	n.s.	n.s.	2.64	–	7.42	–	–	–
	Q.HD.LC-5H.3	5H.3	59.03	314-559	11_20487	6.23	0.00	1	0.77	1.18	1.62	n.s.	n.s.	n.s.	1.48	20.28	4.84	–	–	–
	Q.HD.LC-7H.2	7H.2	15.97	1213-1959	11_10056	7.31	0.00	1	n.s.	−1.29	−1.09	n.s.	n.s.	n.s.	–	24.11	2.19	–	–	–
1000-grain weight	Q.TGW.LC-2H	2H	47.93	6384-866	11_21096	3.87	0.00	1	−1.22	−1.15	−0.55	n.s.	n.s.	n.s.	11.06	12.73	3.81	–	–	–
	Q.TGW.LC-5H.3	5H.3	66.97	ABC04352-pHv108-01	11_11092	3.17	0.00	0	−0.68	−0.68	−0.68	−0.68	−0.68	−0.68	3.47	4.48	5.86	4.54	7.45	3.42
	Q.TGW.LC-6H	6H	48.08	4258-1498	11_20720	3.12	0.00	0	0.70	0.70	0.70	0.70	0.70	0.70	3.63	4.70	6.14	4.76	7.81	3.58
	Q.TGW.LC-7H.2	7H.2	15.97	1213-1959	11_10056	4.03	0.00	1	0.75	n.s.	0.90	n.s.	n.s.	n.s.	4.23	–	10.12	–	–	–
grain weight per plant	Q.GWP.LC-2H	2H	10.74	5880-2547	11_21015	8.00	0.00	1	−0.21	−0.15	−0.17	−0.09	−0.07	−0.13	41.04	31.71	28.63	13.56	8.98	14.77
Length of main stem	Q.LSt.LC-1H.2	1H.2	32.59	5048-1685	11_10729	6.73	0.00	0	−1.88	−1.88	−1.88	−1.88	−1.88	−1.88	9.40	15.95	7.03	10.39	14.16	8.23
	Q.LSt.LC-2H	2H	14.78	7747-1056	11_21261	10.14	1.90	1	−2.00	n.s.	n.s.	−3.36	−2.70	n.s.	10.71	–	–	33.26	29.25	–
	Q.LSt.LC-3H.1	3H.2	36.57	2346-318	11_10283	3.60	0.00	1	n.s.	1.26	n.s.	n.s.	n.s.	2.62	–	7.16	–	–	–	16.10
Number of productive tillers per plant	Q.NPT.LC-2H-1	2H	10.74	5880-2547	11_21015	13.90	0.00	1	0.45	−0.15	0.13	0.29	n.s.	0.10	45.52	6.55	7.26	30.47	–	4.71
	Q.NPT.LC-2H-2	2H	74.42	6117-1507	11_10823	3.47	1.90	1	n.s.	n.s.	n.s.	n.s.	n.s.	−0.17	–	–	–	–	–	13.82
	Q.NPT.LC-2H-3	2H	109.05	5088-59	11_10731	4.47	0.00	0	−0.15	−0.15	−0.15	−0.15	−0.15	−0.15	5.02	6.26	9.29	7.86	14.49	11.00
	Q.NPT.LC-5H.3	5H.3	72.01	1306-408	11_10080	5.71	0.00	0	0.17	0.17	0.17	0.17	0.17	0.17	6.83	8.52	12.63	10.68	19.70	14.95
	Q.NPT.LC-6H	6H	41.21	4191-268	11_20707	2.50	0.00	0	0.10	0.10	0.10	0.10	0.10	0.10	2.22	2.76	4.10	3.47	6.39	4.85
Grain weight per main spike	Q.GWSm.LC-2H-1	2H	10.74	5880-2547	11_21015	14.08	0.00	1	−0.10	−0.06	−0.11	−0.11	−0.05	−0.07	61.23	38.72	63.96	71.98	49.71	26.55
	Q.GWSm.LC-2H-2	2H	113.18	3000-1074	11_10404	2.40	0.00	1	0.03	n.s.	0.03	n.s.	n.s.	0.03	5.08	1.32	5.53	–	–	4.42
	Q.GWSm.LC-2H-3	2H	138.98	1344-930	11_10085	3.99	−5.92	1	n.s.	n.s.	n.s.	n.s.	−0.03	n.s.	–	–	–	–	17.30	–
	Q.GWSm.LC-4H	4H	45.08	3127-273	11_20482	2.44	0.00	0	0.02	0.02	0.02	0.02	0.02	0.02	2.24	4.45	2.03	2.14	8.88	1.79
	Q.GWSm.LC-5H.3	5H.3	61.54	ConsensusGBS0138-2	11_11448	3.96	0.00	0	−0.02	−0.02	−0.02	−0.02	−0.02	−0.02	3.39	6.73	3.07	3.24	13.44	2.70
Number of grains per main spike	Q.NGSm.LC-2H-1	2H	10.74	5880-2547	11_21015	23.66	0.00	1	−2.55	−1.92	−2.31	−2.98	−1.14	−2.04	84.41	70.06	77.10	107.03	66.76	56.85
	Q.NGSm.LC-2H-2	2H	140.95	1344-930	11_10085	4.14	−3.95	1	n.s.	n.s.	n.s.	n.s.	−0.51	0.48	–	–	–	–	13.61	3.14
	Q.NGSm.LC-4H-1	4H	5.91	2533-773	11_10319	4.99	−1.44	1	n.s.	−0.61	−0.56	−0.53	n.s.	n.s.	–	7.15	4.50	3.44	–	–
	Q.NGSm.LC-4H-2	4H	28.79	14765-388	11_20180	3.92	−1.66	1	n.s.	n.s.	n.s.	n.s.	−0.27	0.41	–	–	–	–	3.64	2.33
	Q.NGSm.LC-4H-3	4H	45.08	3127-273	11_20482	4.19	0.00	0	0.47	0.47	0.47	0.47	0.47	0.47	2.87	4.23	3.19	2.67	11.39	3.03
	Q.NGSm.LC-4H-4	4H	76.48	5245-304	11_10751	5.37	0.00	1	n.s.	n.s.	−0.66	−0.65	n.s.	−0.64	–	–	6.21	5.09	1.03	5.63
	Q.NGSm.LC-5H.3	5H.3	70.25	ConsensusGBS0086-5	11_11441	2.03	−1.25	1	−0.96	n.s.	−0.76	−0.77	n.s.	−0.83	11.84	–	8.43	7.15	–	9.45
	Q.NGSm.LC-6H-1	6H	38.46	2176-891	11_10244	6.63	0.00	1	−1.01	−0.62	−1.09	−0.85	−0.34	−1.26	13.24	7.30	17.23	8.79	5.98	21.72
	Q.NGSm.LC-6H-2	6H	60.09	3773-756	11_20620	4.51	0.00	1	0.47	0.82	0.47	0.85	0.27	0.89	2.90	12.82	3.24	8.70	3.77	10.75
Number of spikelets per main spike	Q.NSSm.LC-2H-1	2H	10.74	5880-2547	11_21015	23.18	0.00	1	−2.56	−2.03	−2.21	−3.29	−1.54	−2.55	80.12	65.48	74.28	114.08	82.47	80.77
	Q.NSSm.LC-2H-2	2H	140.95	1344-930	11_10085	3.43	−3.95	1	n.s.	n.s.	n.s.	n.s.	−0.61	n.s.	–	–	–	–	13.12	–
	Q.NSSm.LC-5H.3-1	5H.3	23.58	10669-188	11_10024	4.00	−3.70	1	−0.66	−0.92	n.s.	−0.93	−0.48	−1.34	5.30	13.48	–	9.04	7.85	22.31
	Q.NSSm.LC-5H.3-3	5H.3	71.5	ConsensusGBS0086-5	11_11441	1.40	0.00	1	−0.75	n.s.	−0.73	n.s.	n.s.	−0.61	6.92	–	8.03	–	–	4.68
	Q.NSSm.LC-6H	6H	38.46	2176-891	11_10244	7.07	0.00	1	−1.08	−0.54	−0.92	−0.64	n.s.	−0.76	14.33	4.67	12.92	4.35	–	7.27
Length of main spike	Q.LSm.LC-2H-1	2H	10.74	5880-2547	11_21015	24.73	0.00	1	−0.86	−0.56	−0.78	−1.00	−0.34	−0.76	101.39	64.31	95.69	99.29	38.20	64.78
	Q.LSm.LC-2H-2	2H	113.18	3000-1074	11_10404	2.65	0.00	0	−0.11	−0.11	−0.11	−0.11	−0.11	−0.11	1.82	2.66	2.09	1.31	4.37	1.48
	Q.LSm.LC-3H.1	3H.1	5.68	5945-748	11_21027	2.09	−1.89	0	0.26	0.26	0.26	0.26	0.26	0.26	9.02	13.22	10.39	6.53	21.72	7.36
	Q.LSm.LC-4H	4H	45.08	3127-273	11_20482	4.67	0.00	0	0.17	0.17	0.17	0.17	0.17	0.17	4.02	5.89	4.63	2.91	9.68	3.28
	Q.LSm.LC-5H.3	5H.3	59.03	314-559	11_20487	14.80	0.00	1	−0.19	−0.21	−0.25	−0.46	n.s.	−0.55	4.77	8.67	9.59	21.32	–	34.44
	Q.LSm.LC-6H-1	6H	30.02	1588-537	11_10129	6.70	0.00	1	−0.14	n.s.	n.s.	−0.16	0.09	−0.25	2.75	–	–	2.55	2.61	6.79
	Q.LSm.LC-6H-2	6H	60.09	3773-756	11_20620	4.92	0.00	1	0.13	0.19	0.16	0.23	0.10	0.31	2.31	7.40	3.85	5.25	3.53	10.58
	Q.LSm.LC-6H-3	6H	72.83	2968-1066	11_10400	4.13	0.00	0	0.24	0.24	0.24	0.24	0.24	0.24	8.11	11.89	9.35	5.87	19.54	6.62
	Q.LSm.LC-7H.2	7H.2	15.97	1213-1959	11_10056	6.61	0.00	0	−0.21	−0.21	−0.21	−0.21	−0.21	−0.21	5.81	8.51	6.69	4.20	13.99	4.74
Grain weight per lateral spike	Q.GWSl.LC-1H.2-1	1H.2	27.76	3786-2204	11_20625	1.01	0.00	0	0.02	0.02	0.02	0.02	0.02	0.02	4.88	8.33	5.40	10.25	12.40	3.99
	Q.GWSl.LC-1H.2-2	1H.2	32.59	5048-1685	11_10729	5.52	0.00	1	−0.05	−0.05	−0.04	n.s.	n.s.	n.s.	19.89	32.18	12.80	–	–	–
	Q.GWSl.LC-2H-1	2H	10.74	5880-2547	11_21015	13.05	0.00	1	−0.08	−0.04	−0.05	−0.03	−0.02	−0.04	60.41	30.83	30.59	23.11	10.00	12.60
	Q.GWSl.LC-2H-2	2H	124.23	4241-445	11_20715	4.35	1.46	1	0.03	0.03	0.03	n.s.	n.s.	n.s.	10.55	13.44	10.11	–	–	–
Number of grains per lateral spike	Q.NGSl.LC-2H-1	2H	10.74	5880-2547	11_21015	19.03	0.00	1	−2.16	−1.31	−1.54	−1.48	−0.94	−1.43	73.12	44.55	64.41	58.53	35.28	39.42
	Q.NGSl.LC-2H-2	2H	124.23	4241-445	11_20715	3.98	1.46	1	0.54	0.78	0.43	n.s.	n.s.	n.s.	4.61	15.88	5.08	–	–	–
	Q.NGSl.LC-2H-3	2H	140.95	1344-930	11_10085	3.12	−3.95	0	−0.55	−0.55	−0.55	−0.55	−0.55	−0.55	4.68	7.72	8.12	8.02	11.82	5.80
	Q.NGSl.LC-3H.1	3H.1	28.16	4593-2007	11_10672	2.24	−1.74	1	0.68	0.70	0.60	n.s.	n.s.	n.s.	7.20	12.64	9.91	–	–	–
Number of spikelets per lateral spike	Q.NSSl.LC-2H	2H	10.74	5880-2547	11_21015	19.69	0.00	1	−2.08	−1.28	−1.34	−1.82	−1.19	−1.63	64.61	35.51	46.95	58.10	41.50	41.71
	Q.NSSl.LC-6H-1	6H	38.46	2176-891	11_10244	2.06	0.00	0	−0.53	−0.53	−0.53	−0.53	−0.53	−0.53	4.13	5.98	7.24	4.88	8.14	4.33
	Q.NSSl.LC-6H-2	6H	59.65	5187-752	11_20892	5.46	0.00	1	0.36	0.70	0.54	0.67	n.s.	0.73	1.94	10.68	7.56	7.90	–	8.37
Length of lateral spike	Q.LSl.LC-2H	2H	10.74	5880-2547	11_21015	20.68	0.00	1	−0.68	−0.43	−0.41	−0.82	−0.50	−0.68	84.33	58.61	42.83	99.56	73.11	81.43
	Q.LSl.LC-5H.3	5H.3	61.54	ConsensusGBS0138-2	11_11448	9.29	0.00	1	−0.17	−0.19	−0.23	−0.30	n.s.	−0.34	5.29	11.55	13.80	12.94	–	20.43
	Q.LSl.LC-6H	6H	72.83	2968-1066	11_10400	5.56	0.00	0	0.27	0.27	0.27	0.27	0.27	0.27	13.02	22.44	18.35	10.37	20.36	12.45
	Q.LSl.LC-7H.2	7H.2	15.97	1213-1959	11_10056	5.79	0.00	0	−0.20	−0.20	−0.20	−0.20	−0.20	−0.20	7.06	12.17	9.95	5.62	11.04	6.75

(a) QTL x E — 1- significant, 0 – not significant

(b) Additive effects: negative—alleles increasing trait value from Lubuski. positive—alleles increasing trait value from CamB

(c) Percentage of variance computed as the ratio of variance for effect and variance in given year; values larger than 100 % indicate overestimation of additive effect in the analysis based on six environments in comparison to individual analyses

(d) QTL strength measured by the statistic − Log10(P-value) with P-values for Wald test computed in accumulated analysis of variance

n.s. – not significant

### QTLs for earliness and yield-forming traits

Four QTLs for heading date (HD) were found on chromosomes 2H (Q.HD.LC-2H), 3H (Q.HD.LC-3H.1), 5H (Q.HD.LC-5H.3) and 7H (Q.HD.LC-7H.2). In the vicinity of the Q.HD.LC-2H, 12 QTLs for plant architecture, spike morphology and grain yield were detected (Q.GWSl.LC-2H-1, Q.GWSm.LC-2H-1, Q.GWP.LC-2H, Q.HD.LC-2H, Q.LSl.LC-2H, Q.LSm.LC-2H-1, Q.NGSl.LC-2H-1, Q.NGSm.LC-2H-1, Q.NPT.LC-2H-1, Q.NSSl.LC-2H, Q.NSSm.LC-2H-1 and Q.LSt.LC-2H) for the region on the short arm of chromosome 2H, whereas one QTL (Q.HD.LC-3H.1) was found for the region detected on chromosome 3H. On chromosome 5H, close to the Q.HD.LC-5H.3, were found QLSm.LC-5H.3, QLSl.LC-5H.3 and QGWSm.LC-5H.3 and also three QTLs were identified in the vicinity of the Q.HD.LC-7H.2: Q.LSm.LC-7H.2, Q.LSl.LC-7H.2 and Q.TGW.LC-7H.2.

**Fig. 2:**
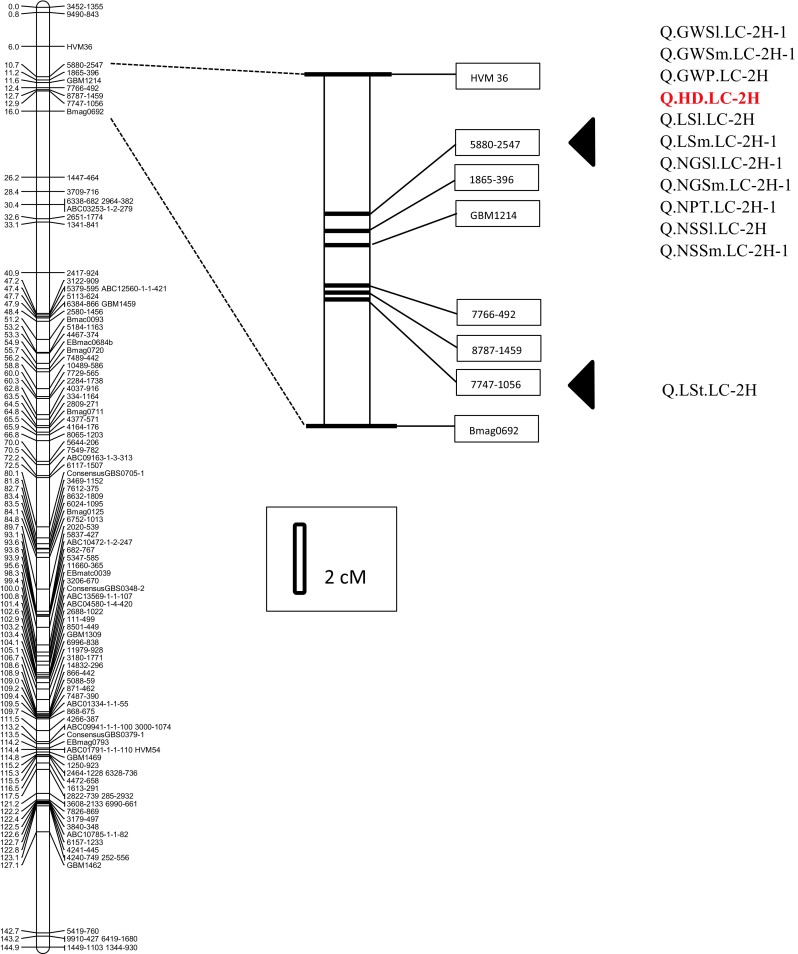
QTLs identified in “hot spot” region on chromosome 2H

For HD the major was QTL on chromosome 2H, which showed the most significant effect and explained a large proportion of the phenotypic variation. This QTL was mapped at the marker 5880–2547 at the position of 10.74 cM (Fig. 2). In almost all environments the alleles from the Syrian parent reduced days to heading; DII 2012 was an exception to this rule, and in this environment the percentage of explained variation was a low (9.55 %).

**Fig. 3:**
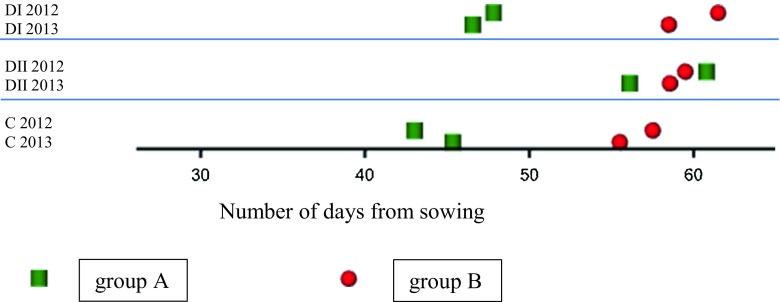
Schematic representation of mean heading dates observed for two subgroups of RILs in different water regimes and years. Groups A, B –homozygotes G/G (CamB) and A/A (Lubuski) at SNP 5880-2547 located in linkage group 2H at 10.74 cM

According to SNP 5880–2547 segregation RILs were divided into two subgroups – early heading (group A – allele from CamB) and late heading plants (group B – allele from Lubuski). The different developmental pattern for these subgroups was noticed in the stress conditions. An extreme delay in heading was observed for early heading lines in DII conditions (Fig. 3).

In the region of Q.HD.LC-2H, marked by SNP 5880–2547 (10.14 cM) and 7747–1056 (14.78 cM), QTLs for all yield-related traits, except TGW, were found (Fig. 2). In all cases they appeared to be the most significant QTLs for a particular trait, with the LogP statistic ranging from 8.00 for GWP to 24.73 for LSm. All of them showed a significant interaction with environment, but the sign of the allelic effects was consistent over environments. For yield-forming traits, except NPT, alleles contributed by Lubuski increased the traits.

Q.HD.LC-3H.1 on chromosome 3H at SNP 10353–119 showed a minor positive effect contributed by a Syrian parent allele. That QTL explained only 2.64–7.42 % of phenotypic variance, and its additive effect was significant only in DI 2012 and C 2012. No QTL associated with other traits was found in this region (Table 3).

The Q.HD.LC-5H.3 located on the linkage group 5H.3 at SNP 314–559 with CamB allele causing later heading was significant only in 2012. In the region of this QTL, marked by SNP 314-559 and ConsensusGBS0138-2, QTLs for LSm, GWSm and LSl were also found, with alleles from Lubuski increasing the trait values, and the variance explained from 0.5 to 34.4 % (Table 3).

The Q.HD.LC-7H.2 was detected on chromosome 7H at SNP 1213–1959. It explained from 2.19 to 24.11 % of the phenotypic variation, with the Lubuski allele increasing the number of days from sowing to heading. Effect of that QTL was significant only in 2012 in DII and C conditions. At the same position QTL for TGW (Q.TGW.LC-7H.2) was localised and significant also only in 2012. That QTL explained 4.23-10.12 % of the phenotypic variance and allele contributed by Lubuski reduced the TGW. Near Q.HD.LC-7H.2 the QTLs for LSm and LSl were also found. These QTLs (Q.LSm.LC-7H.2 and Q.LSl.LC-7H.2, both with stabile effects) explained 4.20–13.99 % and 5.62–12.17 % of the phenotypic variation, respectively. Both QTLs were characterised by Lubuski alleles increasing the traits (Table 3).

### Functional annotation of QTLs

For a biological interpretation of the QTL regions identified on the basis of linkage analysis, we refer to the annotation of SNP using the Ensembl Plants barley gene space according with the approach used in Mikołajczak et al. (2016). The analysis revealed two of the main GO biological processes (defense response and protein ubiquitination) overrepresented in the annotation genes for traits: grain weight per main spike, grain weight per plant and length of main spike (Table 4). The largest number of genes (15) annotated with the previously mentioned terms was noticed for “protein ubiquitination”. Functional annotation analysis also showed six other biological processes overrepresented in the annotation of genes occurring in the QTL regions (defense response, lipid transport, metabolic process, oxidation-reduction process, protein phosphorylation, protein ubiquitination, response to oxidative stress, transmembrane transport (Table 5).

**Table 4:** GO biological process terms over represented in the annotation of genes occurring in the QTL regions for a trait

GO term	Trait	QTL ID	No. of genes	List of genes (MLOC)
Defense response	grain weight per main spike	Q.GWSm.LC-2H-2	7	MLOC_14076
		Q.GWSm.LC-2H-2		MLOC_76088
		Q.GWSm.LC-2H-2		MLOC_5583
		Q.GWSm.LC-2H-2		MLOC_69392
		Q.GWSm.LC-2H-2		MLOC_25677
		Q.GWSm.LC-2H-2		MLOC_16581
		Q.GWSm.LC-5H.3		MLOC_77713
Defense response	grain weight per plant	Q.GWP.LC-2H	1	MLOC_69399
Protein ubiquitination	length of main spike	Q.LSm.LC-2H-2	15	MLOC_40031
		Q.LSm.LC-2H-2		MLOC_54978
		Q.LSm.LC-2H-2		MLOC_679
		Q.LSm.LC-2H-2		MLOC_60024
		Q.LSm.LC-2H-2		MLOC_8581
		Q.LSm.LC-2H-2		MLOC_81408
		Q.LSm.LC-2H-2		MLOC_63051
		Q.LSm.LC-2H-2		MLOC_39480
		Q.LSm.LC-2H-2		MLOC_63511
		Q.LSm.LC-2H-2		MLOC_38436
		Q.LSm.LC-3H.1		MLOC_69418
		Q.LSm.LC-3H.1		MLOC_68550
		Q.LSm.LC-3H.1		MLOC_68553
		Q.LSm.LC-3H.1		MLOC_64722
		Q.LSm.LC-3H.1		MLOC_3103
		Q.LSm.LC-3H.1		MLOC_6570
		Q.LSm.LC-5H.3		MLOC_4665
		Q.LSm.LC-6H-1		MLOC_58751
		Q.LSm.LC-6H-1		MLOC_68356

**Table 5:** GO biological process terms over represented in the annotation of genes occurring in the regions of QTLs

GO biological process term	QTL ID	Total number of genes in the QTL region	Number of genes annotated with the term	List of genes (MLOC)
Defense response	Q.GWSm.LC-2H-2	246	6	MLOC_14076, MLOC_76088, MLOC_5583, MLOC_69392, MLOC_25677, MLOC_16581
	Q.NGSm.LC-2H-2	147	2	MLOC_63489, MLOC_20004
Lipid transport	Q.LSt.LC-1H.2	227	5	MLOC_70721, MLOC_52372, MLOC_55993, MLOC_64544, MLOC_46285
	Q.NPT.LC-5H.3	77	2	MLOC_42618, MLOC_16268
	Q.NGSm.LC-5H.3	162	3	MLOC_42618, MLOC_57612, MLOC_38396
Metabolic process	Q.HD.LC-2H	47	4	MLOC_44360, MLOC_12202, MLOC_51066, MLOC_25950
	Q.LSt.LC-1H.2	227	8	MLOC_70129, MLOC_70743, MLOC_70745, MLOC_17987, MLOC_5359, MLOC_62584, MLOC_67176, MLOC_14711
Oxidation-reduction process	Q.NGSm.LC-2H-1	47	3	MLOC_52158, MLOC_44360, MLOC_25950
Protein phosphorylation	Q.GWSm.LC-2H-1	47	5	MLOC_39533, MLOC_61989, MLOC_38009, MLOC_80756, MLOC_63818
	Q.LSm.LC-3H.1	86	13	MLOC_55753, MLOC_55752, MLOC_36868, MLOC_36867, MLOC_10272, MLOC_40282, MLOC_6370, MLOC_67657, MLOC_55684, MLOC_66868, MLOC_73709, MLOC_14788, MLOC_42962
Protein ubiquitination	Q.LSm.LC-2H-2	246	9	MLOC_40031, MLOC_54978, MLOC_679, MLOC_60024, MLOC_8581, MLOC_81408, MLOC_63051, MLOC_39480, MLOC_63511
Response to oxidative stress	Q.NSSm.LC-2H-2	147	5	MLOC_54892, MLOC_54893, MLOC_65477, MLOC_72076, MLOC_57664
Transmembrane transport	Q.LSl.LC-7H.2	71	6	MLOC_2098, MLOC_76366, MLOC_36691, MLOC_12388, MLOC_44081, MLOC_9846

## Discussion

The present study examined the mapping population derived from the cross Lubuski × Cam/B1/CI08887//CI05761 and special attention was given to earliness. The Syrian genotype, when compared to the European cultivar, was generally characterised by earlier heading and lower yield. Drought stress conditions caused the reduction of the studied traits (with the exceptions: HD and NPT). Grain yield was the most decreased under drought stress applied at the flag leaf stage. This was due to reduced spikelets and grain numbers per spike. These results are in agreement with the results reported by Zinselmeier et al. (1999) and Samarah et al. (2009) who demonstrated the impact of drought during the flowering period on grain yield.

The effect of water scarcity on yield varies depending on the plant development stage. This is why we noted different mean values for traits observed in drought I and drought II. It is noteworthy that in DI and DII treatments plants were observed to have more tillers than in the well-watered conditions. It may be explained by the emergence of new tillers during re-watering period. A similar phenomenon has also been noticed in other works, e.g. by Aspinall et al. (1964) and Loss and Siddique (1994), but in most studies a significant decrease of the number of productive tillers under drought conditions has been observed (Samarah 2005; Shirazi et al. 2010; Tsenov et al. 2015).

In our study, stress conditions caused a delay in heading. These findings are in agreement with other studies (Winkel et al. 1997; Wopereis et al. 1996; Farooq et al. 2011) but, on the other hand, our results are also in contrast to the results showed by Desclaux and Romet (1996), Slafer et al. (2005) and Richards (2006), where the drought conditions caused an acceleration of plant growth and development. In the present studies the highest delay in the appearance of the heading was triggered by drought stress conditions II, especially for early heading lines. This phenomenon can be associated with the survival strategies of this group of plants, which were, in general, at an advanced stage of development at the time of stress application. The results of the study confirmed the assumption that the drought escape can be an effective strategy only when a plant has completed its life cycle before the environment conditions become unfavourable.

### QTLs for earliness

Earliness affects the plant adaptation to the environmental changes and it is a trait affected by numerous QTLs (Yano et al. 2000; Sameri et al. 2011). In our study, four QTLs for earliness were detected on chromosomes 2H, 3H, 5H and 7H. The localisation of these QTLs on barley chromosomes is consistent with previously identified QTLs (Hayes et al. 1993; Laurie et al. 1995; Thomas et al. 1995; Bezant et al. 1996; Tinker et al. 1996; Qi et al. 1998; Pillen et al. 2003). Some of them were found in genomic regions that have been reported to harbour genes involved in flowering time regulation. In our study, the main effects were shown by QTL detected on chromosome 2H at SNP 5880–2547 (11_21015). On the short arm of that chromosome the major photoperiod response locus (*Ppd-H1*) which causes early flowering under day length has been mapped (Laurie et al. 1995). The 2HS region association with the earliness was also observed in numerous other studies. Ren et al. (2010) identified three QTLs determining the heading date on chromosomes 2H (and also on 3H and 7H), which is in agreement with our results. QTL analysis of the Steptoe/ Morex population conducted by Mansour et al. (2014) revealed the QTL also located at SNP 11_21015. All these findings support the notion that the region on the short arm of chromosome 2H is tightly associated with heading date. SNP 11_21015 has been mapped close to markers 12_30871 and 12_30872 (Muñoz-Amatriaín et al. 2011) which are SNPs in *Ppd-H1*. Borrás-Gelonch et al. (2012), following a series of experiments involving environments with artificially extended photoperiod, reported the two QTLs affected earliness on chromosome 2H. The regions on 2H were also the main determinants of heading date in autumn-sown experiments conducted using mapping populations grown under Mediterranean conditions (Moralejo et al. 2004; Cuesta-Marcos et al. 2008). Comadran et al. (2011) reported five QTLs for heading date (located on 1H, 2H, 3H and 5H). Their research revealed that the highest effect was shown by two QTLs detected in the centromeric region of 2H, where another major gene affecting heading date (*eam6*) had previously been reported (Cuesta-Marcos et al. 2009).

Although our study was based on the analysis of a population derived from spring barley parents, QTL analysis revealed some associations with chromosome regions harbouring genes related to vernalisation requirements. Vernalisation response has been shown to be strongly influenced by photoperiod (Roberts et al. 1988; Wang et al. 2010b). Epistatic interaction among major loci of vernalisation response, photoperiod reaction and earliness *per se* may be responsible for the fact that a large number of genomic regions have been identified as determinants of heading date (Karsai et al. 2001). In our study Q.HD.LC-3H.1 was detected on chromosome 3H at SNP 10353–119 and in the vicinity (0.24 cM) of microsatellite Bmag603. Wang et al. (2010b) revealed that a flowering time candidate gene (*HvFT2*) had been located 3 cM from this SSR marker. Another locus connected with heading date was found in our study on chromosome 5H at SNP 314–559 positioned at 59.03 cM (Q.HD.LC-5H.3). This QTL was located in a similar position as QTL for heading date reported by Marquez-Cedillo et al. (2001) and Thomas et al. (1995) and the vernalisation response gene (*Vrn-H1*) found by Laurie et al. (1995). According to Muñoz-Amatriaín et al. (2011), *Vrn-H1* contains SNP 12_30883 and is mapped on the long arm of chromosome 5H between SNPs 11_21247 (7639-122) and 11_11080 (ABC03900-1-2-406), the latter being located in the consensus map used in our studies in the distance of 0.6 cM from SNP 314-559. Our data also showed that in the vicinity of SNP 314–559 another SNP 7523–440 (11_21241) was located, which was linked to the locus *Vrn-H1* in the study conducted by Malosetti et al. (2011).

### QTLs for agronomic traits

Several yield-related QTLs have been mapped to the short arm of chromosome 2H, including plant height (Karsai et al. 1997), kernel weight (Han and Ullrich 1993), number of seeds per spike (Kjaer et al. 1991) and tiller number (Eshghi et al. 2011). In the present study, QTLs with large effects for yield, plant height, number of productive tillers, length of spike, spikelet number and number and weight of grain were found near SNP 5880–2547 on chromosome 2H. Results obtained in numerous studies have shown that loci associated with the length of spikes are placed on all the barley chromosomes (Hori et al. 2003; Sameri et al. 2006; Baghizadeh et al. 2007; Wang et al. 2010a, b). The localisation of the QTL for earliness on chromosome 2H coincided with QTLs for spike morphology. The QTL for the length of the main spike (LSm) was discovered in genomic regions associated with earliness, except the one which was found on chromosome 3H. Interestingly, the QTL for LSm was found in our study both on chromosome 5H (Q.LSm.LC-5H.3) and on chromosome 7H (Q.LSm.LC-7H.2), where were identified regions related to heading stage. In the present study, the QTL for number of grains per main spike was mapped at the marker 5880–2547 on chromosome 2H. The QTLs affecting the number of grains per spike on chromosome 2H have been reported by Mohammadi and Baum (2008) and Mehravaran et al. (2014), and in our investigation, SNP 5880–2547 was also the nearest marker for QTLs related to grain weight per main and lateral spike and grain weight per plant. These results are in agreement with the findings of Peighambari et al. (2005) who found the QTL for grain yield on chromosome 2H. In other studies QTLs for grain yield were identified on almost all the barley chromosomes (Cuesta-Marcos et al. 2009; Mansour et al. 2014; Mehravaran et al. 2014). Stem length is an important morphological character directly linked with the productive potential of barley plants. In the present study, we did not detect any QTL for the length of stem close to SNP 5880–2547 associated with earliness. However, the QTL analysis revealed Q.LSt.LC-2H at SNP 7747-1056, 2.2 cM shifted from from SNP 5880–2547. In the region of QTL for HD detected on 3H no QTL for stem length was found. It should be noted that on 3H *sdw1/denso* locus causing reduction of plant height was localized and several studies revealed that this locus may also be associated with flowering time (Barua et al. 1993; Laurie et al. 1994; Bezant et al. 1996; Kuczyńska et al. 2013, 2014).

In our experiment, two QTLs associated with the numbers of spikelets per spikes (Q.NSSm.LC-2H and Q.NSSl.LC-2H) were found on chromosome 2H at the SNP 5880–2547. These results are in agreement with the QTL localisation previously reported by Li et al. (2005) and Baghizadeh et al. (2007). Additionally, these authors revealed QTLs for these traits also on chromosomes 1H, 5H and 7H.

In the current study, we have identified locus associated with the number of productive tillers on 2H (Q.NPT.LC-2H) at the same position as the main QTL for heading date — position 10.7 cM). Tiller number is a key component of barley grain yield (Sakamoto and Matsuoka 2004). Fertile tillers contribute significantly to grain yield improvement, but those tillers without fertile spikes decrease the harvest index (Mäkelä and Muurinen 2011). In our study we noticed an increase in the number of productive tillers triggered by drought conditions which could be explained by the secondary tiller development process, commonly observed in the field conditions (Aspinall et al. 1964).

### QTLs related to drought

A recent study revealed that QTLs related to drought stress tolerances were placed on chromosome 2H and 5H (Fan et al. 2015). The pivotal importance of the genomic regions for drought tolerance was also reported by Mehravaran et al. (2014) on chromosomes 2H, 5H and 7H. The authors suggested that these regions may be used as an important target for improving drought tolerance of barley.

The association of heading date and drought tolerance has been reported by Xu et al. (2005), Araus et al. (2002), Kigel et al. (2011), Schmalenbach et al. (2014). Similar results were obtained in the present studies. We identified QTLs related to heading date on chromosomes 2H, 5H and 7H, where QTLs for drought tolerance have been reported in other studies. QTLs connected with yield structure were found near QTLs identified for earliness, which is also in agreement with other studies (Wang et al. 2010a; Honsdorf et al. 2014; Mansour et al. 2014; Mehravaran et al. 2014).

In the present study, early heading barley plants did not realise the drought escape strategy, and other mechanisms also associated with water scarcity tolerance appeared to be ineffective. On the other hand, we observed an increase in productive tillers forming after drought during re-watering, especially in the Syrian parent. As early heading lines tend to have low quality yield, the enhancement of productive tillers seems to be a promising strategy.

### Functional annotations

The overrepresentation of genes annotated as “defense response” for traits: grain weight per main spike and grain weight per plant did not allow for an unambiguous interpretation. This GO term was descript by QuickGO (http://www.ebi.ac.uk/QuickGO) as “reactions, triggered in response to the presence of a foreign body or the occurrence of an injury, which result in restriction of damage to the organism attacked or prevention/recovery from the infection caused by the attack”, which can be assigned to a every type of plant reaction associated with biotic or abiotic stresses. Noteworthy, the second type of overrepresented GO term was related to protein ubiquitination as “the process in which one or more ubiquitin groups are added to a protein”. Ubiquitin is well established as the major modifier of signalling in eukaryotes. The main characteristic of ubiquitination is the conjugation of ubiquitin onto lysine residues of acceptor proteins (Stone and Callis 2007). In most cases, the targeted protein is degraded by the 26S proteasome, the major proteolysis machinery in eukaryotic cells. The ubiquitin–proteasome system is responsible for removing most abnormal peptides and short-lived cellular regulators. This allows cells to respond rapidly to intracellular signals and changing environmental conditions. These types of biological processes are crucial to sustain cellular functions under drought. In *Arabidopsis thaliana* more than 1400 genes encode components of the ubiquitin/26S proteasome (Ub/26S) pathway (Smalle and Vierstra 2004). Approximately 90 % of these genes encode subunits of the E3 ubiquitin ligases, which confer substrate specificity to the pathway. This mechanism can be observed in the gibberellin-dependent signalling pathway that regulates the flowering process (Cheng et al. 2004). Gibberellins (GAs), one kind of endogenous growth regulator, play an essential role not only in reproductive development of plants but also in stem and spike growth regulation (Kumar et al. 2003; Tyagi and Singh 2006; Janowska and Andrzejak 2010). Moreover, treatment of GA causes stem elongation, expansion and proliferation and cell wall thickening increased cell division and cell elongation (Taiz and Zeiger 1998). Similar processes may be observed in spike growth and development. Our plant material was differential in terms of spike length both in well-watered and drought conditions, which may suggest that the effect of GA can be a major factor related to spike growth irrespective of irrigation conditions.

The annotation of QTL regions by genes occurring in the projected support intervals showed the six other biological processes, one of which may play a key role in the drought stress. “Response to oxidative stress” was annotated for five genes occurring in the regions of identified QTLs. Prolonged drought stress results in oxidative damage due to the over production of reactive oxygen species (ROS) (Smirnoff 1993). ROS seem to have a dual effect under drought stress conditions that depend on their overall cellular amount. If kept at relatively low levels they are likely to function as components of a stress-signalling pathway, triggering stress defense/acclimation responses. However, when reaching a certain level of phytotoxicity, ROS become damaging, initiating unwell-watered led oxidative cascades that harm cellular membranes and other cellular components resulting in oxidative stress and eventually cell death (Dat et al. 2000) and—as a consequence—the wilting process noticed in water scarcity conditions.

## Conclusions

Four QTLs for HD were detected on chromosomes 2H, 3H, 5H and 7H. Hence, the major was QTL located on the short arm of 2H chromosome at SNP marker 5880–2547, in the vicinity of *Ppd-H1* gene. In the region of SNP 5880–2547 QTLs associated with plant architecture, spike morphology and grain yield were localised. The present study showed that the earliness allele from the Syrian parent, as introduced into the genome of a European variety could result in an improvement of barley yield performance under drought conditions. Screening barley cultivars for growth duration under terminal drought stress is needed to evaluate drought escape in barley grown under such conditions. In order to use these QTLs for improvement of agronomic traits, further complementary studies in different environments and genetic contexts should be performed.

## Electronic supplementary material

Below is the link to the electronic supplementary material.

ESM 1 (DOCX 71 kb)

ESM 2 (DOCX 38 kb)

ESM 3 (DOCX 69 kb)

ESM 4 (DOCX 36 kb)
