# Supplementary material for: QTLs for earliness and yield-forming traits in the Lubuski × CamB barley RIL population under various water regimes
Source: J Appl Genet. 2016 Aug 9;58(1):49–65. doi: 10.1007/s13353-016-0363-4 (PMC5243898; doi:10.1007/s13353-016-0363-4)
Supplement: Supplementary file 1 — (DOCX 71 kb) [file 13353_2016_363_MOESM1_ESM.docx]

QTLs for earliness and yield-forming traits in the Lubuski × CamB barley RIL population under various water regimes

Piotr Ogrodowicz^a^, Tadeusz Adamski^a^, Krzysztof Mikołajczak^a^, Anetta Kuczyńska^a^, Maria Surma^a^, Paweł Krajewski^a^, Aneta Sawikowska^a^, Andrzej G. Górny^a^, Kornelia Gudyś^b^, Iwona Szarejko^b^, Justyna Guzy-Wróbelska^b^, Karolina Krystkowiak^a,*^

^a^  Institute of Plant Genetics of the Polish Academy of Sciences, Strzeszyńska 34, 60-479 Poznań, Poland

^b^ Department of Genetics, Faculty of Biology and Environmental Protection, University of Silesia, Jagiellońska 28, 40-032 Katowice, Poland

*Corresponding authors:

Tel.: (+48 61) 65 50 224; e-mail: [kkry@igr.poznan.pl](mailto:kkry@igr.poznan.pl)


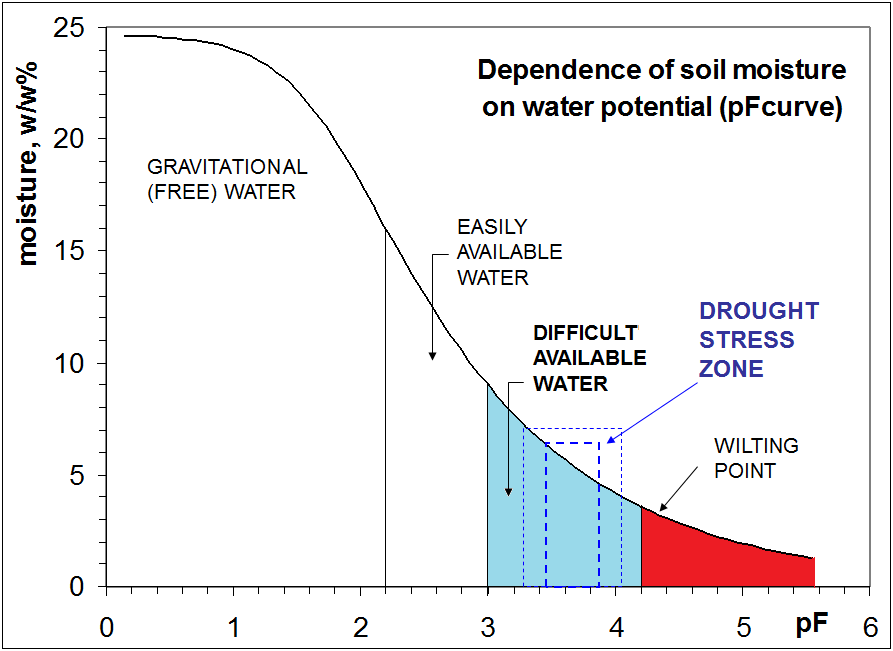


ESM_1. Water retention curve. The pF value is defined as a logarithm of the pressure p (expressed in centimeters of water head) necessary for water removal from soil capillaries
