# Supplementary material for: QTLs for earliness and yield-forming traits in the Lubuski × CamB barley RIL population under various water regimes
Source: J Appl Genet. 2016 Aug 9;58(1):49–65. doi: 10.1007/s13353-016-0363-4 (PMC5243898; doi:10.1007/s13353-016-0363-4)
Supplement: Supplementary file 2 — (DOCX 38 kb) [file 13353_2016_363_MOESM2_ESM.docx]

QTLs for earliness and yield-forming traits in the Lubuski × CamB barley RIL population under various water regimes

Piotr Ogrodowicz^a^, Tadeusz Adamski^a^, Krzysztof Mikołajczak^a^, Anetta Kuczyńska^a^, Maria Surma^a^, Paweł Krajewski^a^, Aneta Sawikowska^a^, Andrzej G. Górny^a^, Kornelia Gudyś^b^, Iwona Szarejko^b^, Justyna Guzy-Wróbelska^b^, Karolina Krystkowiak^a,*^

^a^  Institute of Plant Genetics of the Polish Academy of Sciences, Strzeszyńska 34, 60-479 Poznań, Poland

^b^ Department of Genetics, Faculty of Biology and Environmental Protection, University of Silesia, Jagiellońska 28, 40-032 Katowice, Poland

*Corresponding authors:

Tel.: (+48 61) 65 50 224; e-mail: [kkry@igr.poznan.pl](mailto:kkry@igr.poznan.pl)

ESM_2. Agronomic traits observed in the greenhouse experiment and the description

| Trait (unit) | Phenotype description | Abbrev. |
| --- | --- | --- |
| Heading date (heading) | Number of days from sowing to emergence of inflorescence (spike) from the flag leaf (51 BBCH), assessed when spikes emerged on at least 50% of plants | HD |
| 1000-grain weight (g) | Average weight of 1000 grains, calculated as average of 1000 * average weight of one grain for 20 spikes in a pot | TGW |
| Grain weight per plant (g) | Average weight of grain collected from one plant, calculated as average of measurements of grain weight for 10 plants | GWP |
| Length of main stem (cm) | Average of measurements of length of stem from ground level to the end of spike (without awns) for 10 main stems in a pot | LSt |
| Number of productive tillers per plant | Average number of tillers with spikes, calculated for 10 mature plants in a pot | NPT |
| Grain weight per main spike (g) | Weight of grain collected from one spike of the main stem - average for 10 grain main spikes | GWSm |
| Number of grains per main spike | Number of grains collected from one spike of main stem - average for 10 main spikes in a pot | NGSm |
| Number of spikelets per main spike | Number of spikelets in spike of main stem-average e for 10 main spikes in a pot | NSSm |
| Length of main spike (cm) | Length of spike from main stem-average for 10 main spikes in a pot (without awns) | LSm |
| Grain weight per lateral spike (g) | Weight of grain from spike of lateral stem - average from a lateral spike for 10 lateral spikes | GWSl |
| Number of grains per lateral spike | Number of grains collected from spike of lateral stem - average for 10 lateral spikes in a pot | NGSl |
| Number of spikelets per lateral spike | Number of spikelets per spike of lateral stem - average for 10 lateral spikes in a pot | NSSl |
| Length of lateral spike (cm) | Length of spike from lateral stem - average for 10 lateral spikes in a pot (without awns) | LSl |
