# Supplementary material for: QTLs for earliness and yield-forming traits in the Lubuski × CamB barley RIL population under various water regimes
Source: J Appl Genet. 2016 Aug 9;58(1):49–65. doi: 10.1007/s13353-016-0363-4 (PMC5243898; doi:10.1007/s13353-016-0363-4)
Supplement: Supplementary file 3 — (DOCX 69 kb) [file 13353_2016_363_MOESM3_ESM.docx]

QTLs for earliness and yield-forming traits in the Lubuski × CamB barley RIL population under various water regimes

Piotr Ogrodowicz^a^, Tadeusz Adamski^a^, Krzysztof Mikołajczak^a^, Anetta Kuczyńska^a^, Maria Surma^a^, Paweł Krajewski^a^, Aneta Sawikowska^a^, Andrzej G. Górny^a^, Kornelia Gudyś^b^, Iwona Szarejko^b^, Justyna Guzy-Wróbelska^b^, Karolina Krystkowiak^a,*^

^a^  Institute of Plant Genetics of the Polish Academy of Sciences, Strzeszyńska 34, 60-479 Poznań, Poland

^b^ Department of Genetics, Faculty of Biology and Environmental Protection, University of Silesia, Jagiellońska 28, 40-032 Katowice, Poland

*Corresponding authors:

Tel.: (+48 61) 65 50 224; e-mail: [kkry@igr.poznan.pl](mailto:kkry@igr.poznan.pl)

ESM_3a. The average days to heading and mean values for agronomic traits for parental cultivars

| Trait | Year | Treatment | Lubuski | | CamB | |
| --- | --- | --- | --- | --- | --- | --- |
|  |  |  | mean | s.e. | mean | s.e. |
| Heading date (days) | 2012 | DI | 63.67 | 0.33 | 43.33 | 0.33 |
|  |  | DII | 61.00 | 0.00 | 42.00 | 0.00 |
|  |  | C | 60.00 | 0.00 | 40.33 | 0.33 |
|  | 2013 | DI | 60.00 | 0.00 | 42.00 | 0.00 |
|  |  | DII | 59.00 | 0.00 | 45.50 | 0.50 |
|  |  | C | 54.00 | 0.00 | 39.00 | 0.00 |
| 1000-grain weight (g) | 2012 | DI | 42.28 | 3.13 | 43.28 | 1.61 |
|  |  | DII | 34.42 | 1.30 | 44.20 | 4.68 |
|  |  | C | 46.08 | 1.68 | 45.66 | 1.22 |
|  | 2013 | DI | 39.14 | 1.94 | 41.15 | 1.27 |
|  |  | DII | 36.57 | 0.04 | 33.14 | 2.39 |
|  |  | C | 45.18 | 2.36 | 43.33 | 0.80 |
| Grain weight per plant (g) | 2012 | DI | 2.74 | 0.04 | 1.89 | 0.04 |
|  |  | DII | 2.55 | 0.10 | 1.94 | 0.29 |
|  |  | C | 3.16 | 0.16 | 2.49 | 0.18 |
|  | 2013 | DI | 1.93 | 0.22 | 1.52 | 0.17 |
|  |  | DII | 1.53 | 0.07 | 0.82 | 0.04 |
|  |  | C | 2.67 | 0.18 | 2.05 | 0.02 |
| Length of main stem (cm) | 2012 | DI | 64.87 | 4.36 | 66.03 | 1.54 |
|  |  | DII | 63.43 | 1.07 | 65.70 | 1.24 |
|  |  | C | 67.63 | 2.93 | 70.97 | 0.67 |
|  | 2013 | DI | 65.50 | 4.50 | 55.50 | 0.50 |
|  |  | DII | 67.00 | 2.00 | 53.00 | 3.00 |
|  |  | C | 69.00 | 4.00 | 74.50 | 2.50 |
| Number of productive tillers per plant | 2012 | DI | 5.13 | 0.15 | 5.73 | 0.30 |
|  |  | DII | 5.67 | 0.35 | 4.50 | 0.55 |
|  |  | C | 4.40 | 0.44 | 4.13 | 0.07 |
|  | 2013 | DI | 3.75 | 0.60 | 4.72 | 0.07 |
|  |  | DII | 4.05 | 0.30 | 2.68 | 0.32 |
|  |  | C | 4.05 | 0.15 | 4.50 | 0.00 |
| Grain weight per main spike (g) | 2012 | DI | 0.75 | 0.08 | 0.52 | 0.01 |
|  |  | DII | 0.59 | 0.03 | 0.74 | 0.04 |
|  |  | C | 0.95 | 0.04 | 0.79 | 0.02 |
|  | 2013 | DI | 0.68 | 0.02 | 0.46 | 0.05 |
|  |  | DII | 0.56 | 0.03 | 0.37 | 0.03 |
|  |  | C | 0.91 | 0.08 | 0.75 | 0.04 |
| Number of grains per main spike | 2012 | DI | 17.50 | 0.55 | 12.11 | 0.52 |
|  |  | DII | 16.43 | 0.27 | 15.80 | 0.86 |
|  |  | C | 19.97 | 0.64 | 16.08 | 1.13 |
|  | 2013 | DI | 16.80 | 0.70 | 10.60 | 0.60 |
|  |  | DII | 14.75 | 0.35 | 10.10 | 0.10 |
|  |  | C | 18.80 | 0.60 | 13.85 | 0.65 |

| Trait | Year | Treatment | Lubuski | | CamB | |
| --- | --- | --- | --- | --- | --- | --- |
|  |  |  | mean | s.e. | mean | s.e. |
| Number of spikelets per main spike | 2012 | DI | 18.67 | 1.34 | 12.83 | 1.23 |
|  |  | DII | 17.93 | 0.88 | 16.33 | 0.33 |
|  |  | C | 21.13 | 0.52 | 16.40 | 0.50 |
|  | 2013 | DI | 18.30 | 0.10 | 11.00 | 0.40 |
|  |  | DII | 16.90 | 1.30 | 12.63 | 0.23 |
|  |  | C | 21.40 | 1.00 | 14.80 | 0.20 |
| Length of main spike (cm) | 2012 | DI | 6.47 | 0.38 | 5.30 | 1.17 |
|  |  | DII | 5.90 | 0.21 | 6.30 | 0.10 |
|  |  | C | 7.70 | 0.06 | 6.03 | 0.15 |
|  | 2013 | DI | 6.30 | 0.20 | 3.65 | 0.25 |
|  |  | DII | 5.90 | 0.50 | 4.39 | 0.19 |
|  |  | C | 7.65 | 0.75 | 5.45 | 0.15 |
| Grain weight per lateral spike (g) | 2012 | DI | 0.67 | 0.09 | 0.49 | 0.04 |
|  |  | DII | 0.48 | 0.03 | 0.57 | 0.20 |
|  |  | C | 0.72 | 0.04 | 0.59 | 0.05 |
|  | 2013 | DI | 0.49 | 0.05 | 0.32 | 0.04 |
|  |  | DII | 0.35 | 0.06 | 0.18 | 0.00 |
|  |  | C | 0.62 | 0.10 | 0.31 | 0.01 |
| Number of grains per lateral spike | 2012 | DI | 15.97 | 1.02 | 11.20 | 0.95 |
|  |  | DII | 14.60 | 0.12 | 13.47 | 0.87 |
|  |  | C | 16.30 | 0.23 | 14.03 | 0.72 |
|  | 2013 | DI | 13.15 | 1.55 | 8.05 | 0.95 |
|  |  | DII | 10.30 | 1.20 | 6.33 | 0.42 |
|  |  | C | 15.20 | 2.80 | 10.55 | 0.35 |
| Number of spikelets per lateral spike | 2012 | DI | 17.67 | 1.65 | 11.93 | 1.27 |
|  |  | DII | 15.80 | 1.03 | 13.67 | 2.37 |
|  |  | C | 17.73 | 1.05 | 14.50 | 0.86 |
|  | 2013 | DI | 15.50 | 0.50 | 8.80 | 0.80 |
|  |  | DII | 13.80 | 1.20 | 9.80 | 2.20 |
|  |  | C | 18.70 | 0.70 | 11.20 | 0.20 |
| Length of lateral spike (cm) | 2012 | DI | 6.07 | 0.56 | 4.00 | 0.12 |
|  |  | DII | 5.13 | 0.09 | 4.38 | 0.65 |
|  |  | C | 6.07 | 0.12 | 4.87 | 0.28 |
|  | 2013 | DI | 5.60 | 0.20 | 2.95 | 0.35 |
|  |  | DII | 4.80 | 0.30 | 3.27 | 0.47 |
|  |  | C | 6.65 | 0.25 | 3.65 | 0.15 |

ESM_3b. The average days to heading and mean values for agronomic traits in RILs

| Trait | Year | Treatment | RILs | | | | |
| --- | --- | --- | --- | --- | --- | --- | --- |
|  |  |  | mean | s.e. | min. | max. | c.v. |
| Heading date (days) | 2012 | I | 58.41 | 0.63 | 44.00 | 65.67 | 10.75 |
|  |  | II | 59.98 | 0.25 | 53.67 | 63.67 | 4.19 |
|  |  | C | 53.91 | 0.74 | 41.67 | 63.33 | 13.80 |
|  | 2013 | I | 55.42 | 0.61 | 43.00 | 62.00 | 11.08 |
|  |  | II | 57.96 | 0.19 | 47.00 | 60.50 | 3.25 |
|  |  | C | 52.92 | 0.52 | 41.00 | 60.00 | 9.79 |
| 1000-grain weight (g) | 2012 | DI | 44.87 | 0.35 | 35.57 | 52.64 | 7.79 |
|  |  | DII | 38.38 | 0.31 | 31.92 | 48.37 | 8.17 |
|  |  | C | 49.18 | 0.27 | 42.34 | 57.65 | 5.58 |
|  | 2013 | DI | 39.33 | 0.32 | 31.56 | 48.31 | 8.06 |
|  |  | DII | 36.90 | 0.24 | 30.24 | 42.66 | 6.61 |
|  |  | C | 43.16 | 0.36 | 35.05 | 52.18 | 8.41 |
| Grain weight per plant (g) | 2012 | DI | 2.73 | 0.03 | 1.86 | 3.50 | 12.32 |
|  |  | DII | 2.39 | 0.03 | 1.66 | 3.14 | 11.59 |
|  |  | C | 3.14 | 0.03 | 2.44 | 3.83 | 10.09 |
|  | 2013 | DI | 1.71 | 0.03 | 1.17 | 2.40 | 14.92 |
|  |  | DII | 1.36 | 0.03 | 0.86 | 2.42 | 18.48 |
|  |  | C | 2.42 | 0.04 | 1.61 | 3.46 | 14.61 |
| Length of main stem (cm) | 2012 | DI | 69.56 | 0.63 | 56.87 | 89.03 | 9.08 |
|  |  | DII | 62.12 | 0.49 | 50.63 | 77.53 | 7.87 |
|  |  | C | 72.37 | 0.70 | 56.97 | 94.57 | 9.71 |
|  | 2013 | DI | 60.68 | 0.59 | 46.00 | 76.00 | 9.75 |
|  |  | DII | 54.05 | 0.50 | 44.00 | 68.00 | 9.32 |
|  |  | C | 72.18 | 0.63 | 57.00 | 86.50 | 8.72 |
| Number of productive tillers per plant | 2012 | DI | 4.66 | 0.06 | 3.33 | 6.60 | 13.78 |
|  |  | DII | 5.48 | 0.06 | 4.20 | 7.40 | 10.81 |
|  |  | C | 3.89 | 0.05 | 2.90 | 5.10 | 12.34 |
|  | 2013 | DI | 3.86 | 0.05 | 2.59 | 5.40 | 13.79 |
|  |  | DII | 3.78 | 0.04 | 2.92 | 4.88 | 10.61 |
|  |  | C | 3.64 | 0.05 | 2.17 | 4.95 | 12.61 |
| Grain weight per main spike (g) | 2012 | DI | 0.79 | 0.01 | 0.44 | 1.11 | 15.69 |
|  |  | DII | 0.61 | 0.01 | 0.42 | 0.84 | 14.31 |
|  |  | C | 1.00 | 0.01 | 0.65 | 1.18 | 13.18 |
|  | 2013 | DI | 0.59 | 0.01 | 0.35 | 0.88 | 21.59 |
|  |  | DII | 0.51 | 0.01 | 0.39 | 0.68 | 12.98 |
|  |  | C | 0.88 | 0.01 | 0.59 | 1.21 | 16.19 |
| Number of grains per main spike | 2012 | DI | 17.24 | 0.27 | 10.13 | 22.03 | 15.63 |
|  |  | DII | 15.64 | 0.22 | 10.83 | 21.00 | 14.18 |
|  |  | C | 19.72 | 0.26 | 13.87 | 24.87 | 13.20 |
|  | 2013 | DI | 14.65 | 0.28 | 8.55 | 20.95 | 19.00 |
|  |  | DII | 13.32 | 0.14 | 9.70 | 16.75 | 10.73 |
|  |  | C | 19.06 | 0.27 | 12.50 | 25.10 | 14.15 |

| Trait | Year | Treatment | RILs | | | | |
| --- | --- | --- | --- | --- | --- | --- | --- |
|  |  |  | mean | s.e. | min. | max. | mean |
| Number of spikelets per main spike | 2012 | DI | 18.34 | 0.28 | 11.40 | 22.73 | 15.14 |
|  |  | DII | 17.34 | 0.25 | 12.40 | 23.60 | 14.31 |
|  |  | C | 20.75 | 0.26 | 14.07 | 25.83 | 12.43 |
|  | 2013 | DI | 15.71 | 0.30 | 9.10 | 23.44 | 18.95 |
|  |  | DII | 15.08 | 0.18 | 11.40 | 19.20 | 11.64 |
|  |  | C | 20.09 | 0.28 | 14.30 | 27.40 | 14.00 |
| Length of main spike (cm) | 2012 | DI | 6.18 | 0.08 | 4.07 | 8.17 | 13.39 |
|  |  | DII | 5.68 | 0.07 | 4.20 | 7.70 | 12.41 |
|  |  | C | 7.31 | 0.08 | 5.63 | 9.77 | 10.64 |
|  | 2013 | DI | 5.17 | 0.10 | 3.15 | 8.22 | 18.79 |
|  |  | DII | 5.11 | 0.06 | 4.15 | 6.78 | 10.92 |
|  |  | C | 6.66 | 0.09 | 4.90 | 9.70 | 13.85 |
| Grain weight per lateral spike | 2012 | DI | 0.68 | 0.01 | 0.41 | 0.91 | 15.31 |
|  |  | DII | 0.53 | 0.01 | 0.36 | 0.73 | 15.38 |
|  |  | C | 0.81 | 0.01 | 0.62 | 1.15 | 12.06 |
|  | 2013 | DI | 0.40 | 0.01 | 0.22 | 0.60 | 17.84 |
|  |  | DII | 0.31 | 0.01 | 0.13 | 0.48 | 21.80 |
|  |  | C | 0.58 | 0.01 | 0.37 | 0.99 | 19.94 |
| Number of grains per lateral spike | 2012 | DI | 15.66 | 0.25 | 9.90 | 19.77 | 15.81 |
|  |  | DII | 14.09 | 0.20 | 10.23 | 18.57 | 14.02 |
|  |  | C | 17.26 | 0.19 | 12.93 | 21.23 | 11.09 |
|  | 2013 | DI | 10.47 | 0.19 | 5.70 | 15.25 | 17.90 |
|  |  | DII | 8.90 | 0.16 | 4.56 | 12.60 | 18.49 |
|  |  | C | 14.58 | 0.22 | 8.20 | 20.35 | 15.37 |
| Number of spikelets per lateral spike | 2012 | I | 16.73 | 0.25 | 10.47 | 20.67 | 15.14 |
|  |  | II | 15.98 | 0.21 | 11.80 | 20.73 | 13.38 |
|  |  | C | 18.14 | 0.20 | 13.13 | 22.33 | 10.88 |
|  | 2013 | I | 12.72 | 0.23 | 7.10 | 18.20 | 18.34 |
|  |  | II | 12.12 | 0.18 | 6.90 | 15.90 | 15.19 |
|  |  | C | 16.49 | 0.25 | 9.40 | 22.70 | 15.22 |
| Length of lateral spike | 2012 | I | 5.61 | 0.07 | 3.77 | 7.40 | 12.99 |
|  |  | II | 5.22 | 0.06 | 4.27 | 7.27 | 10.81 |
|  |  | C | 6.22 | 0.06 | 5.03 | 8.13 | 10.09 |
|  | 2013 | I | 4.34 | 0.08 | 2.60 | 6.70 | 18.44 |
|  |  | II | 4.17 | 0.06 | 2.55 | 5.80 | 14.25 |
|  |  | C | 5.62 | 0.07 | 3.30 | 8.00 | 13.23 |
